# Supplementary figures and images for: circHIPK3 nucleates IGF2BP2 and functions as a competing endogenous RNA
Source: eLife. 2024 Jul 23;13:RP91783. doi: 10.7554/eLife.91783 (PMC11265796; doi:10.7554/eLife.91783)

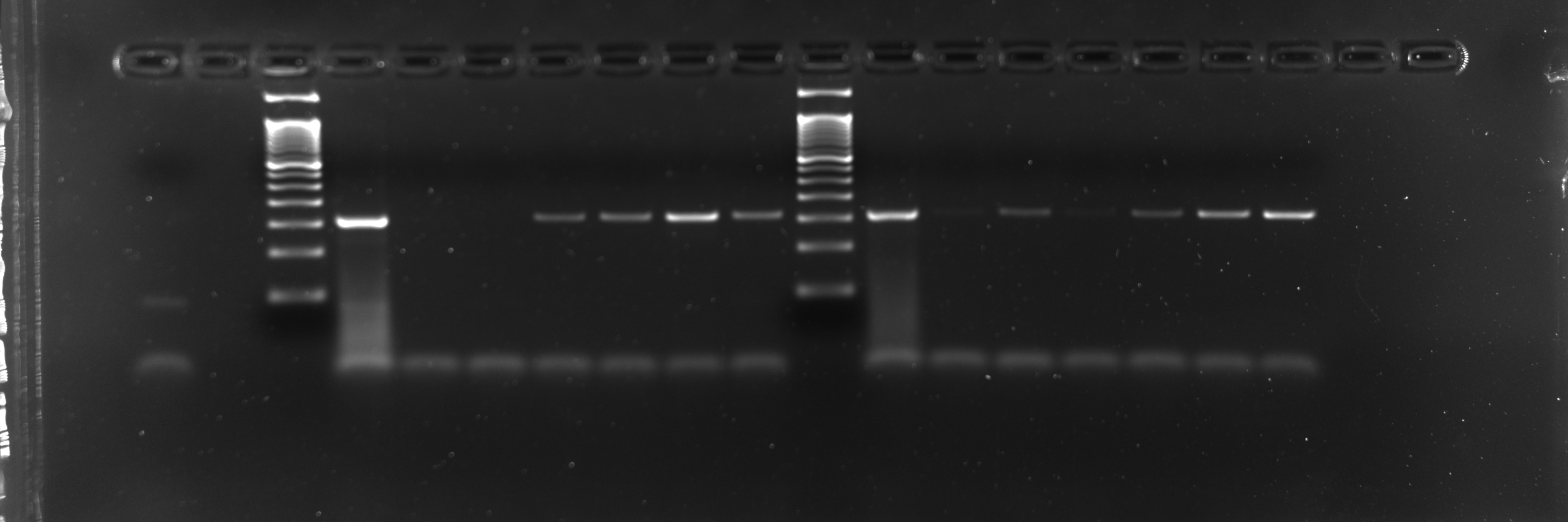

Supplement: Figure 3—source data 1. — Bands included in the main figure are marked. [file elife-91783-fig3-data1.zip › Figure 3 source data/Figure 3 source data.jpg]

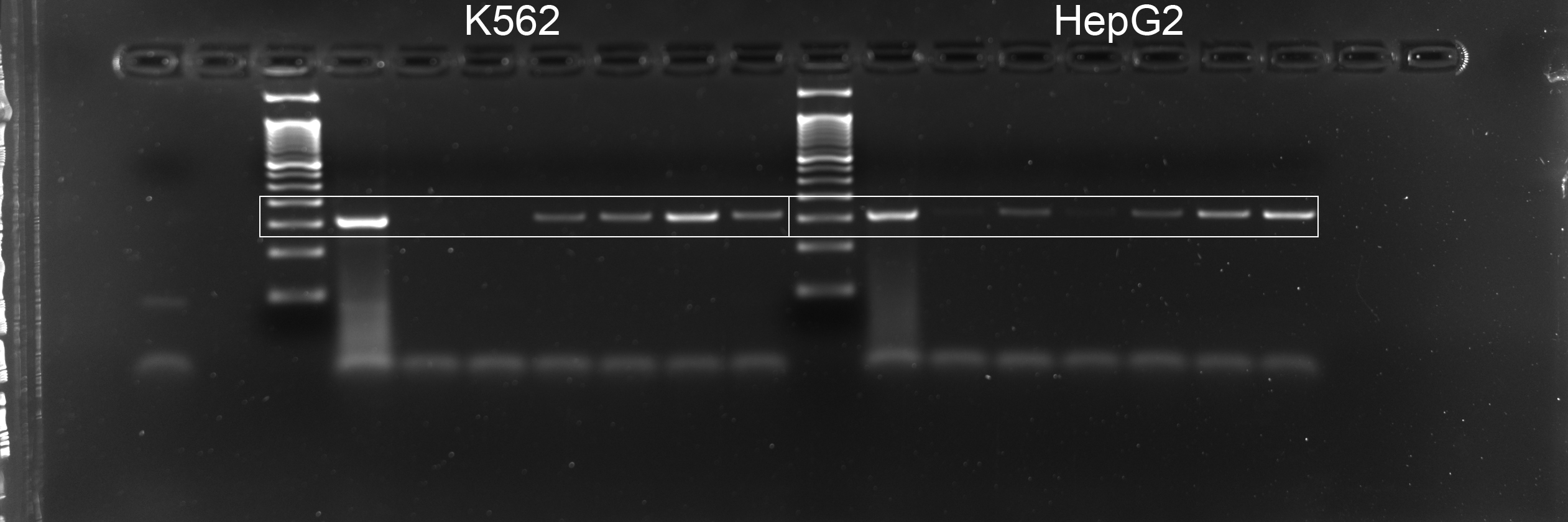

Supplement: Figure 3—source data 1. — Bands included in the main figure are marked. [file elife-91783-fig3-data1.zip › Figure 3 source data/Figure 3 source data_mark.jpg]

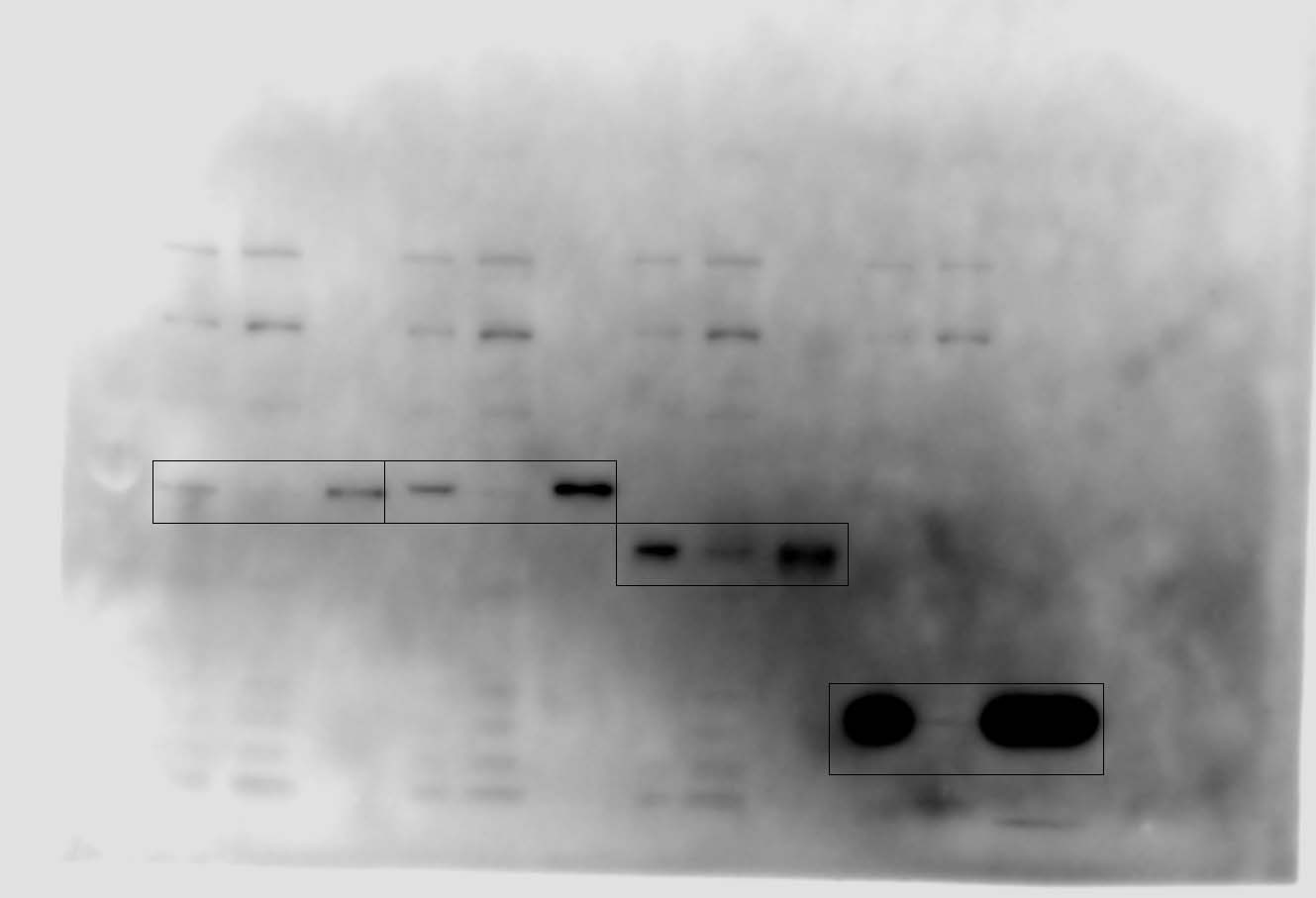

Supplement: Figure 4—figure supplement 1—source data 1. [file elife-91783-fig4-figsupp1-data1.zip › Figure 4 source files/Figure 4 - figure supplement 1 - source data 2_mark.jpg]

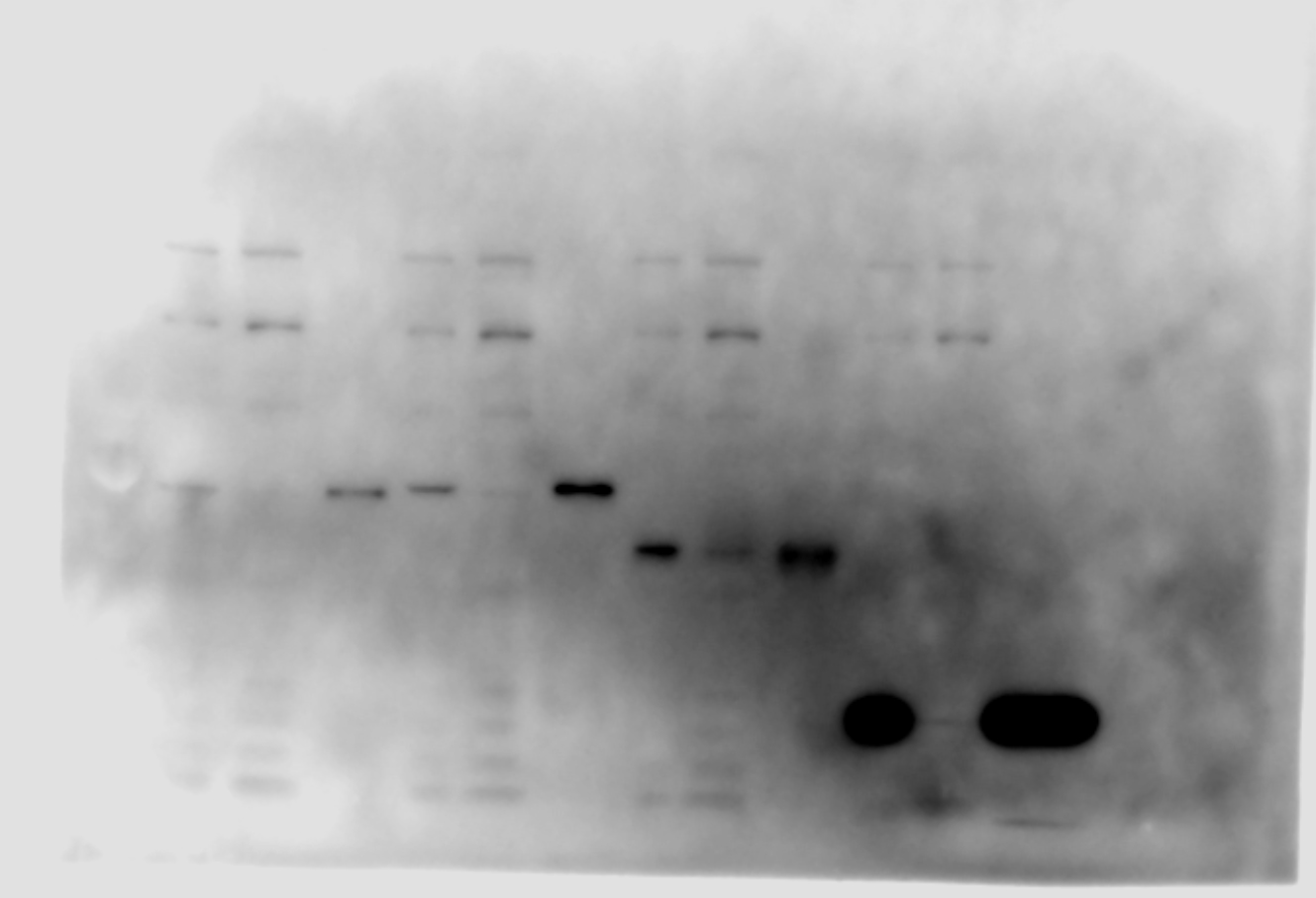

Supplement: Figure 4—figure supplement 1—source data 1. [file elife-91783-fig4-figsupp1-data1.zip › Figure 4 source files/Figure 4 - figure supplement 1 - source data 2.jpg]

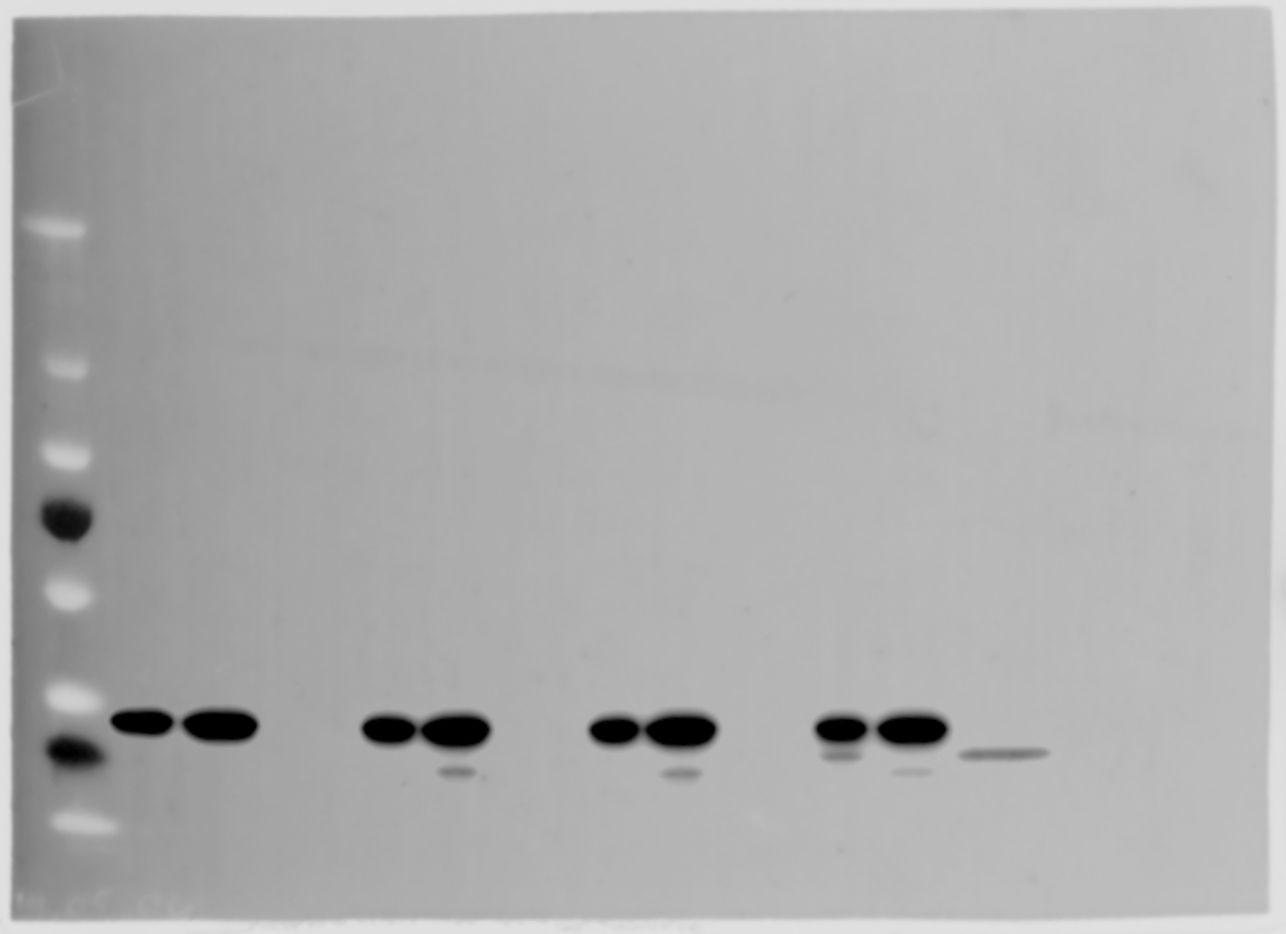

Supplement: Figure 4—figure supplement 1—source data 1. [file elife-91783-fig4-figsupp1-data1.zip › Figure 4 source files/Figure 4 - figure supplement 1 - source data 1.jpg]

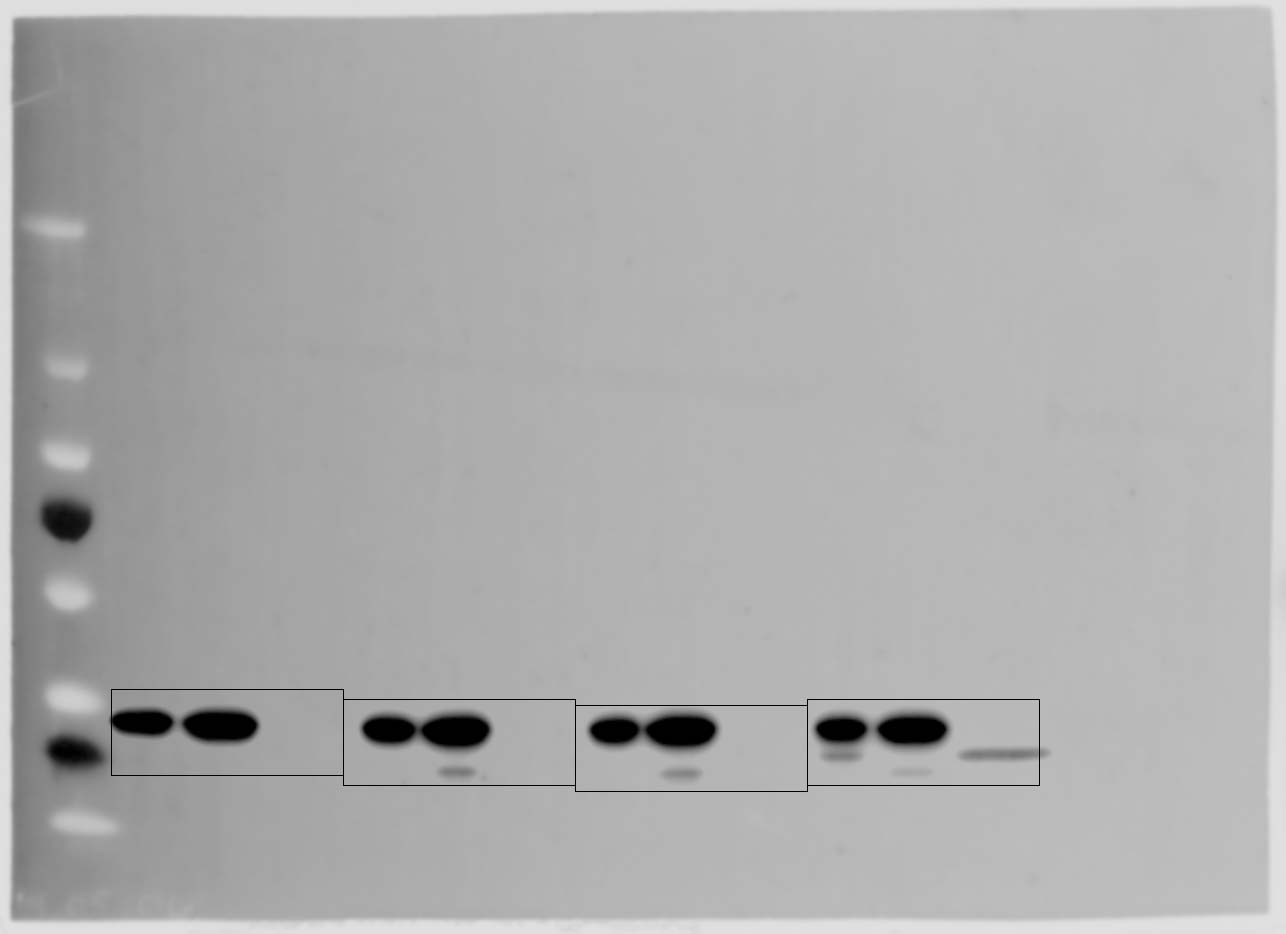

Supplement: Figure 4—figure supplement 1—source data 1. [file elife-91783-fig4-figsupp1-data1.zip › Figure 4 source files/Figure 4 - figure supplement 1 - source data 1_mark.jpg]

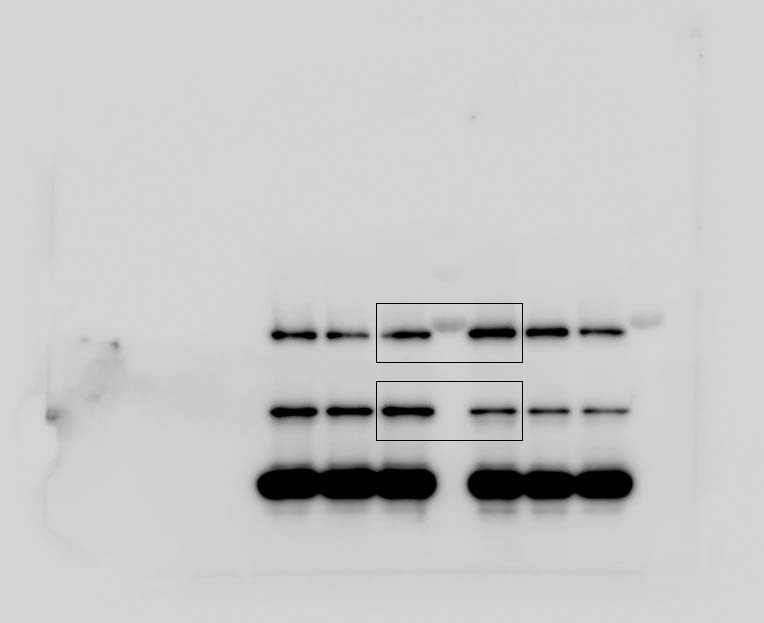

Supplement: Figure 5—source data 1. [file elife-91783-fig5-data1.zip › Figure 5 source files/Figure 5 - source data 1_mark.jpg]

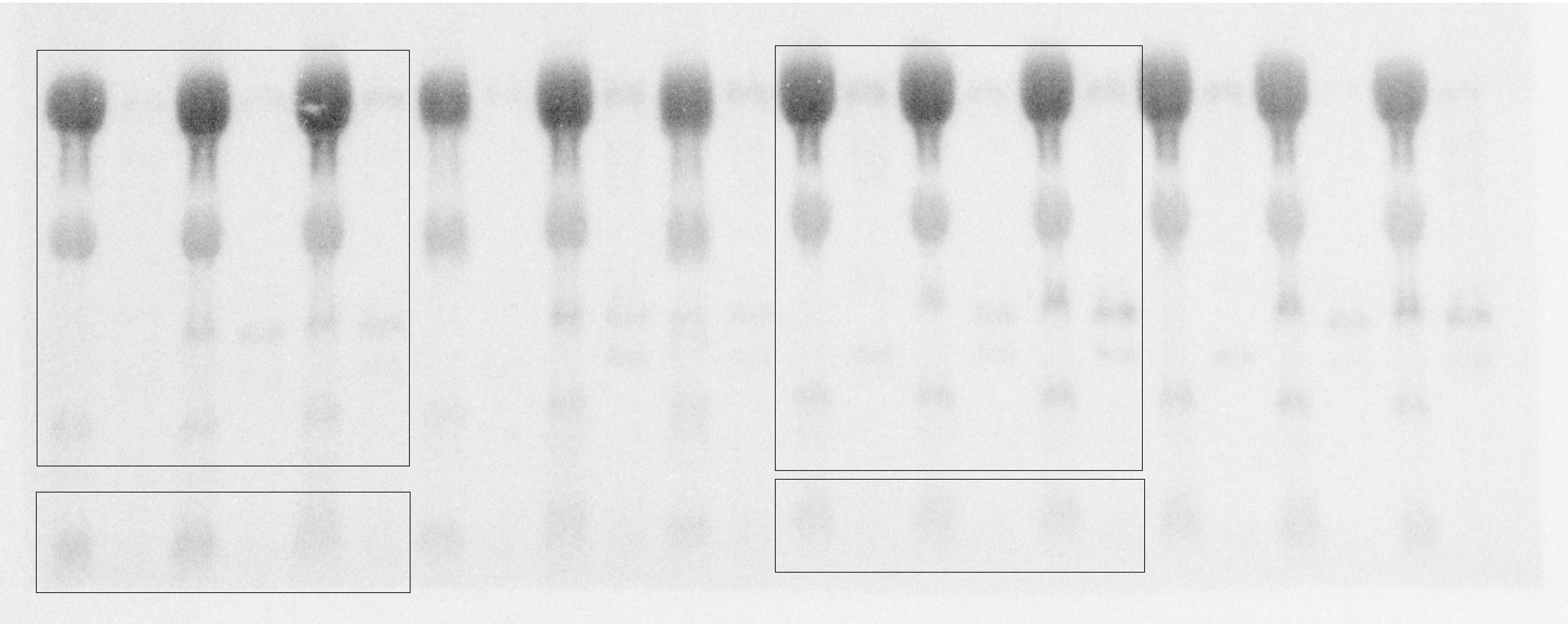

Supplement: Figure 5—source data 1. [file elife-91783-fig5-data1.zip › Figure 5 source files/Figure 5-figure supplement 5H - source data_mark.jpg]

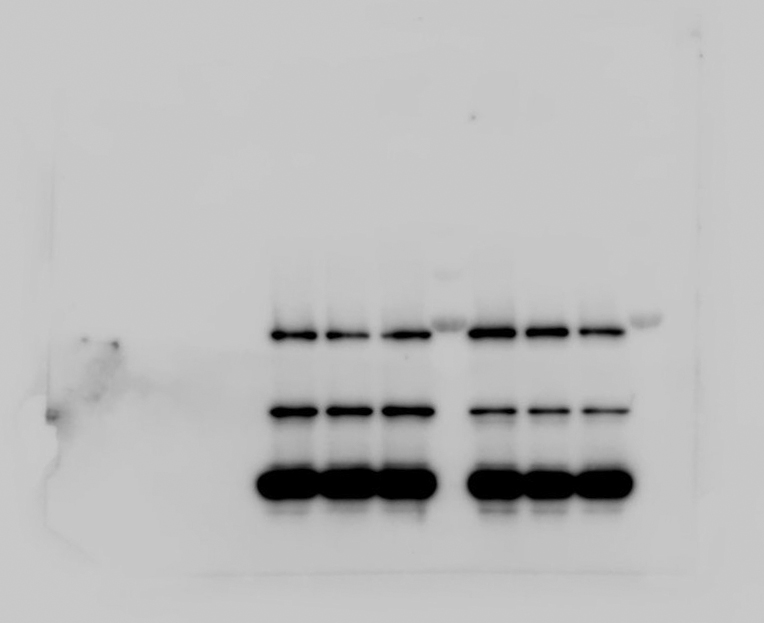

Supplement: Figure 5—source data 1. [file elife-91783-fig5-data1.zip › Figure 5 source files/Figure 5 - source data 1.jpg]

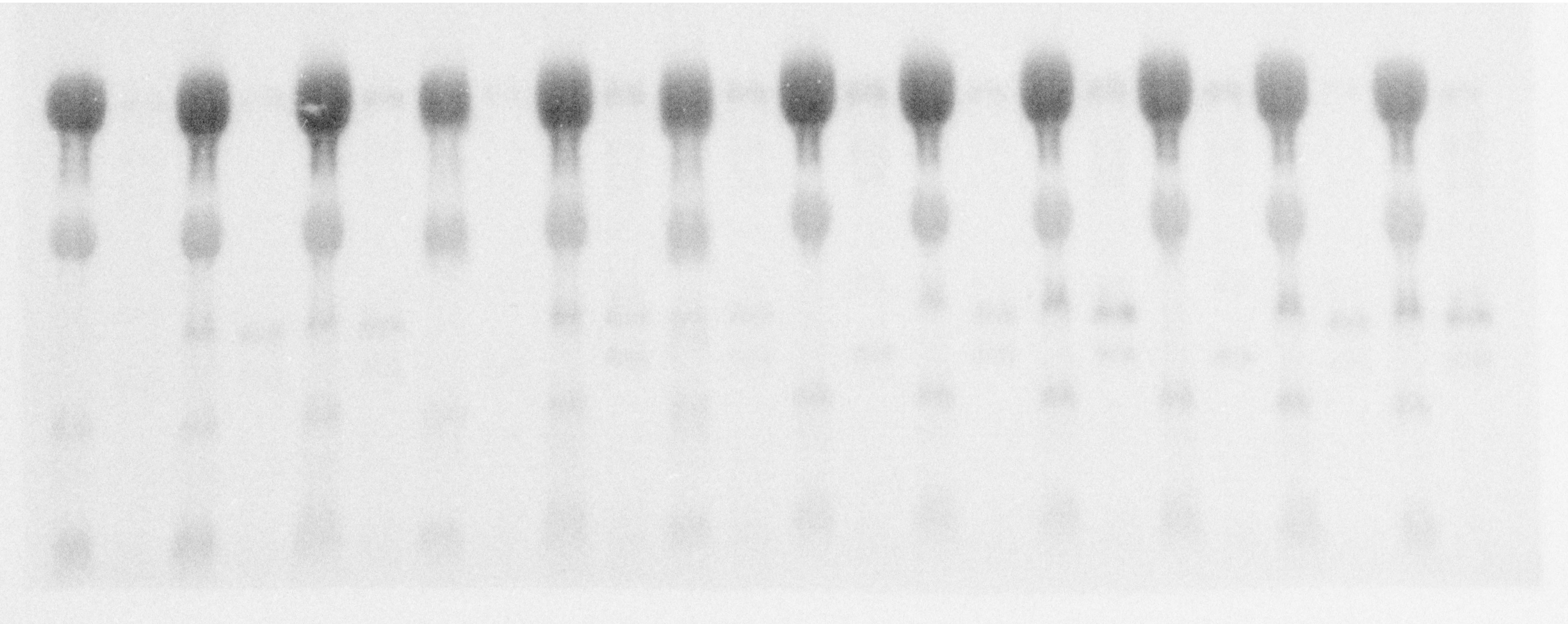

Supplement: Figure 5—source data 1. [file elife-91783-fig5-data1.zip › Figure 5 source files/Figure 5-figure supplement 5H - source data.jpg]

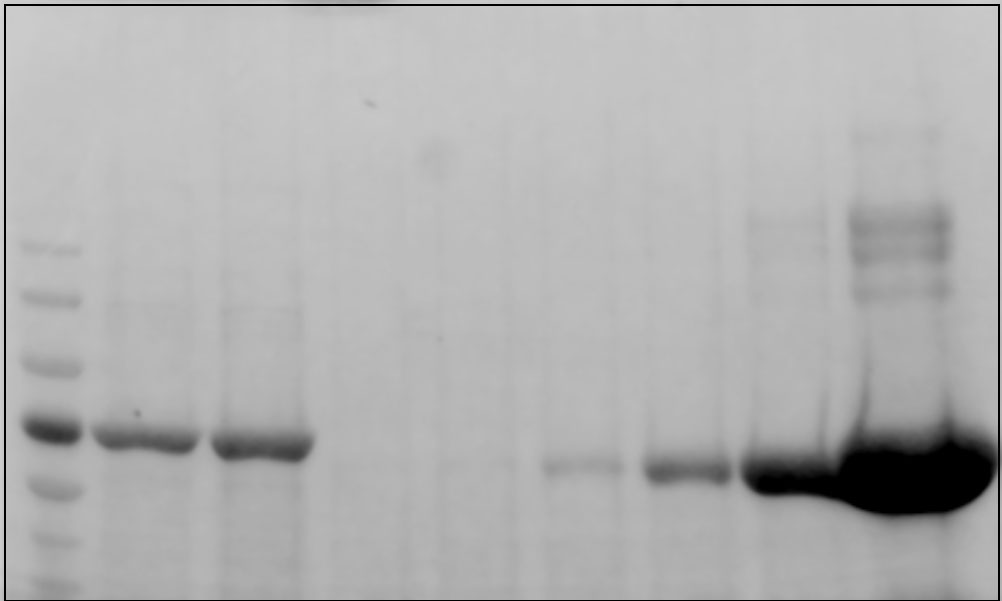

Supplement: Figure 6—figure supplement 1—source data 1. [file elife-91783-fig6-figsupp1-data1.zip › Figure 6 source files/Figure 6-figure supplement 6D - source data_mark.jpg]

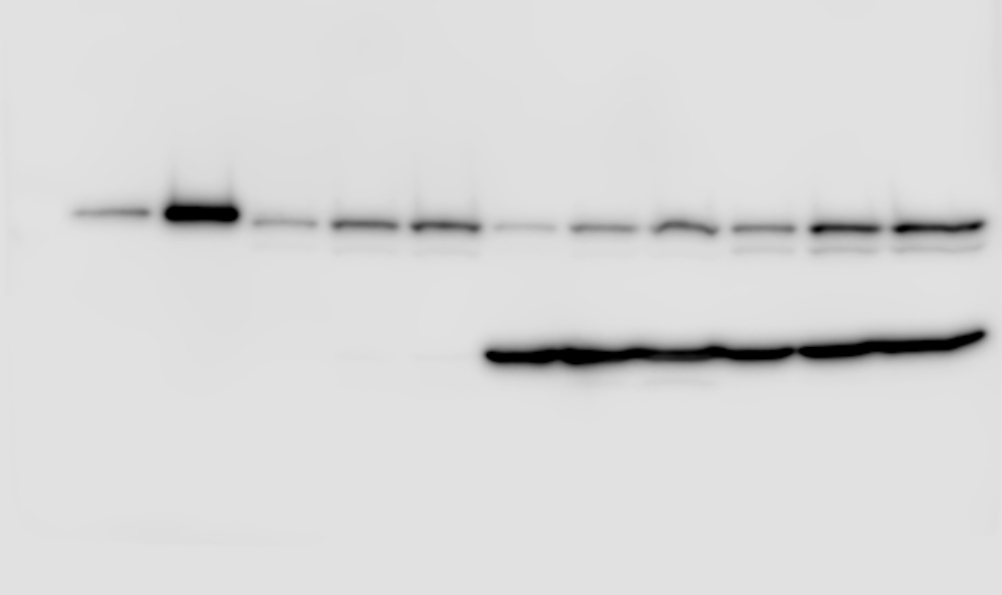

Supplement: Figure 6—figure supplement 1—source data 1. [file elife-91783-fig6-figsupp1-data1.zip › Figure 6 source files/Figure 6-figure supplement 6E - source data.jpg]

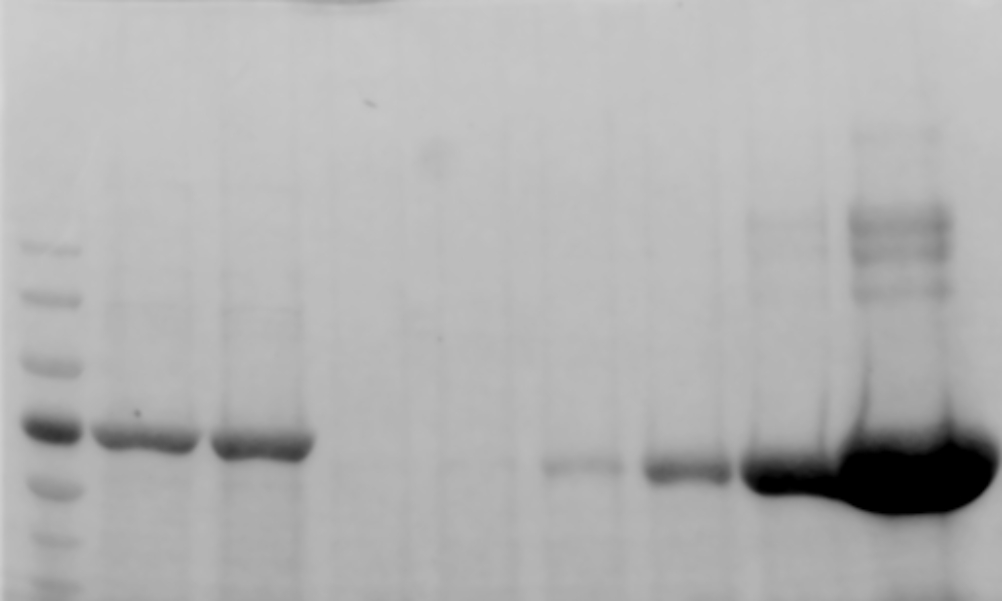

Supplement: Figure 6—figure supplement 1—source data 1. [file elife-91783-fig6-figsupp1-data1.zip › Figure 6 source files/Figure 6-figure supplement 6D - source data.jpg]

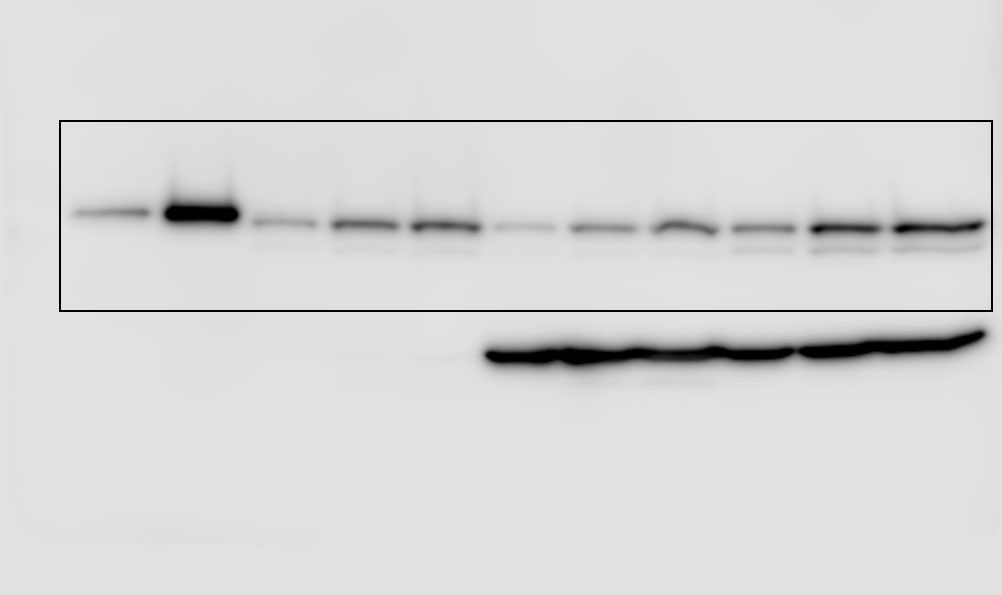

Supplement: Figure 6—figure supplement 1—source data 1. [file elife-91783-fig6-figsupp1-data1.zip › Figure 6 source files/Figure 6-figure supplement 6E - source data_mark.jpg]
